# Learner evaluation of an immersive virtual reality mass casualty incident simulator for triage training

**DOI:** 10.1186/s44247-024-00117-5

**Published:** 2024-09-16

**Authors:** David P. Way, Ashish R. Panchal, Alan Price, Vita Berezina-Blackburn, Jeremy Patterson, Jillian McGrath, Douglas Danforth, Nicholas E. Kman

**Affiliations:** 1 Department of Emergency Medicine, The Ohio State University College of Medicine, 782 Prior Hall, 376 W. 10 Ave, Columbus, OH. 43210 USA; 2 Department of Emergency Medicine, The Ohio State University College of Medicine, 760 Prior Hall, 376 W. 10 Ave, Columbus, OH. 43210 USA; 3 Center for Immersive Media, University of the Arts, 310 S. Broad St, Philadelphia, PA 19102 USA; 4 Advanced Computing Center for the Arts and Design, The Ohio State University, 339B Sullivant Hall, 1813 N. High Street, Columbus, OH 43210 USA; 5 Advanced Computing Center for the Arts and Design, The Ohio State University, 331E Sullivant Hall, 1813 N. High Street, Columbus, OH 43210 USA; 6 Department of Obstetrics & Gynecology, The Ohio State University College of Medicine, 395 W. 12 Ave, Columbus, OH 43210 USA

**Keywords:** Virtual reality, educational, Emergency medical services, Emergency responders, Triage, Education, professional, Mass casualty incidents, Professional competence, Disaster planning, Educational measurement

## Abstract

**Background:**

To minimize loss of life, modern mass casualty response requires swift identification, efficient triage categorization, and rapid hemorrhage control. Current training methods remain suboptimal. Our objective was to train first responders to triage a mass casualty incident using Virtual Reality (VR) simulation and obtain their impressions of the training’s quality and effectiveness.

We trained subjects in a triage protocol called Sort, Assess, Lifesaving interventions, and Treatment and/or Transport (SALT) Triage then had them respond to a terrorist bombing of a subway station using a fully immersive virtual reality simulation. We gathered learner reactions to their virtual reality experience and post-encounter debriefing with a custom electronic survey. The survey was designed to gather information about participants’ demographics and prior experience, including roles, triage training, and virtual reality experience. We then asked them to evaluate the training and encounter and the system’s potential for training others.

**Results:**

We received 375 completed evaluation surveys from subjects who experienced the virtual reality encounter. Subjects were primarily paramedics, but also included medical learners as well as other emergency medical service (EMS) professionals. Most participants (95%) recommended the experience for other first responders and rated the simulation (95%) and virtual patients (91%) as realistic. Ninety-four percent (94%) of participants rated the virtual reality simulator as “excellent” or “good.” We observed some differences between emergency medical service and medical professionals regarding their prior experience with disaster response training and their opinions on how much the experience contributed to their learning. We observed no differences between subjects with extensive virtual reality experience and those without.

**Conclusions:**

Our virtual reality simulator is an automated, customizable, fully immersive virtual reality system for training and assessing personnel in the proper response to a mass casualty incident. Participants perceived the simulator as an adequate alternative to traditional triage and treatment training and believed that the simulator was realistic and effective for training. Prior experience with virtual reality was not a prerequisite for the use of this system.

**Supplementary Information:**

The online version contains supplementary material available at 10.1186/s44247-024-00117-5.

## Background

Throughout the United States (U.S.), mass casualty incidents (MCIs) are increasing in both number and scope [1, 2]. MCIs may be natural or man-made, although incidents involving shootings or explosions are becoming increasingly common [2–4]. As MCIs have increased, there has been a reciprocal focus on training first responders for these types of events [4–6]. MCIs, particularly those involving shootings or explosions, require complex decision making since they involve: security threats, life-threatening injuries, challenging triage and transport decisions, coordination among response personnel and interoperability across diverse agencies [7]. Most MCI training efforts to date can be classified into three paradigms: 1) live, large-scale simulations of mass casualty incidents, cast in temporary settings, that involve the use of manikins and actors; [8–11] 2) tabletop drills that resemble board games; [12] and 3) lectures or presentations on mass casualty response, sometimes followed by facilitated discussions about treating specific patient injuries [13]. These types of simulation platforms provide first responders with methods for increasing knowledge and confidence but are unlikely to provide the sustained practice they need to be able to deliver successful triage and treatment of patients while under the duress of a mass casualty incident [14].

More recently, with the advent of higher-end gaming computers and computer interfaces, virtual reality (VR) systems provide alternative platforms for training individuals in skills required to respond to complex scenarios such as MCIs [15, 16]. Compared to traditional mass casualty incident training methods, VR systems can be programmed to portray the chaotic environment associated with MCIs [17]. Scenarios can be customized to be progressively more challenging through manipulation of noise, debris, and other distractions. Additionally, scenarios can be made more difficult through the addition of more patients who suffer higher degrees of injury acuity. These systems are portable, reusable and allow for continuous practice opportunities for first responders to gradually improve their skills in responding to MCIs [17].

When faced with an MCI, first responders are required to perform two complex tasks. First, they must effectively diagnose the entire scene, which involves sorting and triaging casualty victims into priority groups for transport to hospitals or trauma care centers. Second, they must be able to diagnose and treat those individual patients who are most at risk of losing their life or limb if they are not treated quickly. Both these skills involve clinical reasoning, which is best learned through repetitive, deliberate practice with performance feedback from an instructor [18].

One triage protocol that has been shown to be effective is the Sort, Assess, Lifesaving interventions, and Treatment and/or Transport (SALT) Protocol [19]. This protocol was proposed by a Center for Disease Control (CDC) sponsored working group to standardize triage methods across the U.S. The SALT Protocol is evidence-informed and endorsed by numerous professional organizations including: the American College of Emergency Physicians, American College of Surgeons Committee on Trauma, National Association of EMS Physicians, and National Disaster Life Support Education Consortium [19]. SALT has several advantages over other protocols. First, it can be applied to both pediatric and adult casualties. Second, it saves time by using voice commands to perform an initial global sort. Third, it eliminates the requirement of having to count respirations or check capillary refill, which are challenging tasks in chaotic environments. Finally, SALT allows for more rapid application of life saving interventions (LSIs) such as tourniquets [13].

The primary purpose of this project was to evaluate the use of a VR platform for training first responders in the skills required for disaster response, specifically skills related to SALT triage and treatment of an MCI. We were guided in our design of this VR simulation platform by the foundational elements of Brain-based Learning Theory, which includes the principle that learning complex clinical tasks is maximized when learners can experience immersion in realistic settings [20, 21]. Because little is known about whether learners would find the VR platform to be an acceptable alternative to traditional disaster training platforms, our objective was to gauge learners’ reactions to this fully immersive VR experience with triaging and treating patients in response to a virtual MCI. A secondary purpose was to determine whether prior experience using VR systems was associated with the participant’s perceptions about the quality of the VR training encounter.

## Methods

### Study design, setting and participants

This study was a prospective observational study of learner impressions of a fully automated, fully immersive VR system for training and assessing personnel to effectively respond to an MCI [17]. The system uses a commercially available laptop gaming computer and a Meta Quest 2 VR headset, (Reality Labs, Meta Platforms, Menlo Park, CA. https://www.meta.com/quest/products/quest-2/) and was designed for portability and practical use by agencies responsible for training first responders. We used the system as part of a short program to train learners in implementing the SALT Triage protocol [22].

Training was delivered to Emergency Medical Service (EMS) clinicians: paramedics and EMTs, from agencies throughout Central Ohio. EMS training sessions were held at the agency’s training facilities or in their service bays. We also held SALT Triage training sessions for future and current emergency medicine physicians: medical students, emergency medicine (EM) residents, and EM physicians. Physicians who are boarded in emergency medicine are commonly involved in the training of EMS professionals in disaster medicine skills. Medical learner training was conducted in medical school classrooms. Study participants completed consent forms and were screened for susceptibility to motion sickness (e.g. vertigo, dizziness), a potential side effect of VR. This study was approved by The Ohio State University Institutional Review Board (Columbus, OH, USA).

### SALT triage training

The SALT Triage training was delivered in four parts. First, learners were introduced to the protocol through a brief lecture. Most learners already knew how to deliver life-saving treatments such as application of a tourniquet, so for those groups, we focused attention on the global sort technique and the definitions of each level of triage. More novice learners were also taught how to perform life-saving treatments. Second, each learner entered the VR simulator and participated in an orientation of the system. Third, the learners were provided the opportunity to perform SALT Triage on a simulation scenario that contained eleven patients, all of whom required triage categorization, and more than half required life-saving treatments. Finally, after the learner’s VR encounter, they received feedback through a debriefing session that was guided by a printed report of their performance. During debriefing, learners were offered the opportunity to explain their reasoning behind the decisions they made during the encounter.

### MCI-VR simulator

**Fig. 1:**
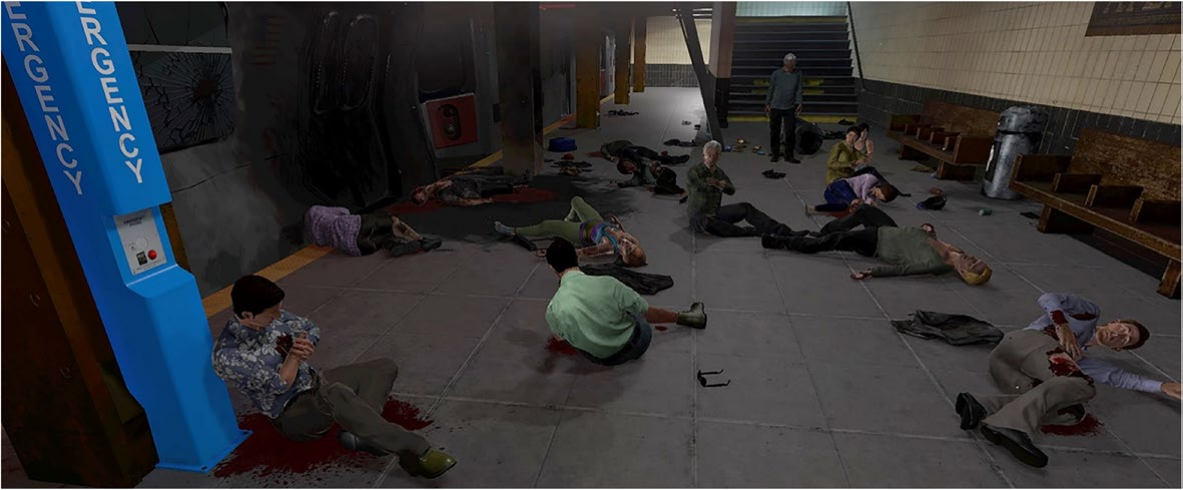
Paramedic participant using the mass casualty incident-virtual reality system

The VR simulator scenario involved the recreation of a terrorist bombing of an underground subway station (Fig. 1). The system is fully described elsewhere but will be briefly summarized here [17]. Participants entered the MCI scene by donning a VR Head-Mounted Display (HMD) and hand-held controllers. While participants could physically walk around the virtual space, the controllers made navigation more efficient through the “teleport” (point to the spot they want to go and click) or “strafe/glide” (small movement) features, which moved them to where they wanted to go without walking. This means that the physical space for the encounter did not have to be the full size of the virtual subway platform, which if converted to actual physical space would be approximately 60 by 30 feet. For safety, participants were provided with a guardian (a research staff member) to ensure that they did not physically venture into physical obstacles.

In VR, participants were equipped with a virtual medical kit. They used the controllers to grab tools and triage tags from the kit, or to take a pulse on a patient. Virtual patient injuries were those commonly suffered by individuals exposed to an explosion. Subsequently, learners could implement various lifesaving interventions (LSIs) including opening an airway of someone struggling to breathe, controlling major hemorrhage with a tourniquet or wound packing, or needle decompressing the chest of a victim who is suffering from a tension pneumothorax. Following completion of the encounter, the participants removed the headset and moved on to the debriefing session.

The MCI-VR System recorded and tabulated essentially every action the learner performed during the simulation encounter to produce a performance report. Faculty evaluators used this performance report to provide feedback to the learner through a post encounter debriefing on their performance.

### Measures

Post-encounter surveys were designed by the researchers primarily to assess learner satisfaction with their VR experience. In addition to items that evaluated features of the system, we asked participants for an overall judgement of its training potential and whether the system encounter supported their learning needs. To gauge whether participants needed prior VR experience to benefit from the encounter, we used ownership of a VR system as a proxy for experience. We asked two open-ended questions about the system’s strengths (effective features of the system), and things we might do to improve it. Members of the VR development team used feedback from the second open-ended item to fine tune the MCI-VR system during Beta testing. The survey also included requests for personal characteristics such as: professional role, level of training, and prior triage experience (Appendix A).

### Analysis

We described our participant characteristics using frequencies and percentages and compared professional groups (EMS vs. Medicine) using Chi Squares (Fisher’s Exact Tests). To assess whether participants’ evaluations of the MCI-VR system features were associated with prior VR experience, we compared those who owned a VR system, to those who did not, using independent t-tests. For items related to how well the MCI-VR encounter contributed to the participants’ learning, and opinions about the utility of the MCI-VR system, we compared EMS to medical professionals using independent t-tests. For statistically significant results, we calculated associated effect sizes: either Cohen’s D for mean statistics or Cramer’s V for Chi Squares. Statistical analyses were performed with IBM-SPSS, Version 29.0.1 (IBM Corp. Released 2022. IBM SPSS Statistics for Windows, Version 29.0.1 Armonk, NY: IBM Corp). To control rates of Type-1 error, we used the Bonferroni method to calculate family-wise levels of significance by dividing the desired *P* = 0.05 level by the number of comparisons within in each set (k/0.05) [23].

We used summative content analysis to code and reduce open-ended survey responses to themes [24–26]. First, one author (ARP) drafted a preliminary coding dictionary based on anticipated survey responses and common responses received in program evaluations. Second, responses to open-ended questions were entered into an Excel workbook (Excel, Version 2211, Redmond, WA, USA). Third, other authors (ARP, DPW, NEK) used word units (i.e., words, word stems, and words phrases with common semantic meanings) to assign codes to each response from the coding dictionary. Comments about strengths and suggestions for improving the VR system were coded separately. Coding assignments were performed iteratively as: 1) the coding dictionary was refined, 2) codes that shared similar underlying concepts were combined, and 3) codes that contained fewer than ten responses were eliminated. Excel continued to be used to process responses into codes until the dataset was effectively reduced to the strongest themes based on the number of times responses appeared.

## Results

Between March 2022 and May 2023 we trained a total of 536 participants using our SALT Training Program which included encounters with the MCI-VR simulation system. Of the individuals trained, 83% (447 of 536) completed post-encounter surveys, however, we excluded 73 individuals who completed less than half of the survey (13.6%; 73 of 536). Subsequently, our final post-encounter survey response was 70% (375 of 536).

### Participant characteristics

Eighty-six percent (86%, 324 of 375) of respondents were EMS professionals (Basic EMTs or Paramedics), and 61% (230 of 374) of respondents considered themselves to be seasoned first responders. One quarter of our subjects (25%, 92 of 374) indicated enthusiasm for computer games, while 17% (64 of 374) reported owning a VR system (Table 1).

**Table 1:** Participant characteristics. Frequencies and percentages of participant demographic characteristics for *N* = 324 EMS participants and *N* = 51 medical participants; with comparisons between groups made with Chi Square (Fisher’s Exact Tests) and Cramer’s V effect sizes (es)

		**EMS** Frequency (%)	**Medicine** Frequency (%)	**Total** Frequency (%)
I am a computer gaming enthusiast	**Yes**	73 (23)	19 (37)	92 (25)
	**No**	251 (77)	32 (63)	283 (75)
I own a VR system	**Yes**	55 (17)	9 (18)	64 (17)
	**No**	269 (83)	42 (82)	311 (83)
I consider myself a seasoned first responder* (es = .44)	**Yes**	226 (70)	4 (8)	230 (61)
	**No**	98 (30)	47 (92)	145 (39)
I have completed the SALT Triage Certificate Training Course	**Yes**	102 (32)	22 (43)	124 (33)
	**No**	222 (68)	29 (57)	251 (67)
I have completed triage training other than SALT Triage Training before* (es = .30)	**Yes**	244 (75)	18 (35)	262 (70)
	**No**	80 (25)	33 (65)	113 (30)
I have completed disaster response training such as those offered by the American Red Cross, FEMA, or the Community Emergency Response Team (CERT)* (es = .28)	**Yes**	196 (60)	10 (20)	206 (55)
	**No**	128 (40)	41 (80)	169 (45)
Number of disaster drills participated in before this encounter	**Mn** **(SD)**	5.2 (14.4)	1.2 (1.8)	2.3 (.90)

Notes: Percentages are based on numbers within professional group (ie. column percentages). Adjusted *P*-values for significance were: k/*P* = .05/7 = .007. Number of disaster drill experiences are presented as means and standard deviations

^*^P ≤ .001; Cramer’s V Effect Sizes can be interpreted as: .10 = small, .30 = medium, .50 = large [27]

To gauge respondents prior triage training and experience, we asked them to identify specific courses they had taken, and the number of large-scale disaster drills in which they had participated. Seventy percent (70%, 262 of 374) of respondents had completed some form of triage training, while only 33% (124 of 374) had been trained specifically in SALT Triage. Fifty-five percent (55%, 206 of 374) had completed a formal disaster response training program; and of that group, the mean number of disaster drills in which they participated was 2.3 drills (Std. Dev. = 0.90) (Table 1).

EMS professionals differed significantly from medical learners regarding training and professional identity as a first responder. Significantly more EMS professionals than medical learners considered themselves to be “seasoned first responders” (*P* ≤ 0.001, es = 0.44). Participants who identified as seasoned first responders were also significantly more likely to have: completed some form of triage training other than SALT (*P* ≤ 0.001, es = 0.29); completed a formal disaster response training course (*P* ≤ 0.001, es = 0.29); and participated in significantly more large-scale disaster drills than those who did not think they were seasoned first responders (*P* ≤ 0.04, es = 0.22). The effect sizes associated with these differences are considered intermediate in size (Table 1).

### Evaluation of VR features

Nearly all participants indicated that the VR orientation helped them to master the use of controllers to navigate the virtual space, (96%, 359 of 374) and subsequently felt sufficiently prepared to enter the MCI-VR subway station scenario (93%, 348 of 374). Almost all participants identified the MCI-VR simulator (95%, 354 of 374) and the patients within as realistic (91%, 340 of 374). They also reported that patients responded to their commands during the encounter (85%, 319 of 374). Participants agreed that the medical kit contained all the tools they needed to field treat patients (91%, 317 of 374), and that tools were easy to use (90%, 335 of 374). We observed no differences in evaluation responses from participants who own VR equipment and those who do not (Table 2).

**Table 2:** VR system evaluation. Means (Mn), standard deviations (SD), frequencies and percentages from participant evaluations of the VR system features comparing *N* = 64 Virtual Reality System Owners to *N* = 310 Non-Owners. Features were evaluated using a 5-point Likert Response Set: (1 = Strongly Disagree, 2 = Disagree, 3 = Disagree/Agree Equally (D/A =), 4 = Agree, 5 = Strongly Agree)

Item	Own VR	N	Mn	SD	Disagree (1-2 s)	D/A (3)	Agree (4-5 s)
The VR simulation exercise was realistic	**Yes**	64	4.38	.58	0	3 (5)	61 (95)
	**No**	310	4.32	.58	1 (.3)	16 (5)	293 (95)
The virtual patients were realistic	**Yes**	64	4.25	.62	0	6 (9)	58 (91)
	**No**	309	4.21	.63	4 (1)	23 (7)	282 (91)
The virtual patients responded to my commands	**Yes**	64	4.06	.85	4 (6)	6 (9)	54 (85)
	**No**	310	4.07	.73	14 (5)	31 (10)	265 (85)
I was adequately prepared to enter the MCI-VR subway station	**Yes**	64	4.39	.63	1 (2)	2 (3)	61 (95)
	**No**	310	4.31	.60	0	23 (7)	287 (93)
I needed more time to acclimate to VR before entering the MCI-VR subway station	**Yes**	64	2.47	1.13	44 (68)	8 (13)	12 (19)
	**No**	309	2.77	1.12	155 (50)	68 (22)	86 (28)
The orientation helped me to master navigation through the MCI-VR subway station	**Yes**	64	4.48	.53	0	1 (2)	63 (98)
	**No**	309	4.37	.59	2 (1)	12 (3)	296 (96)
Navigation throughout the MCI-VR subway station was challenging	**Yes**	64	2.28	1.13	46 (72)	10 (16)	8 (12)
	**No**	310	2.54	1.04	181 (58)	71 (23)	58 (19)
The medical kit contained everything I needed	**Yes**	64	4.17	.75	3 (5)	4 (6)	57 (89)
	**No**	310	4.07	.86	25 (8)	25 (8)	260 (84)
I found it easy to use instruments from the medical kit	**Yes**	64	4.38	.63	0	5 (8)	59 (92)
	**No**	310	4.18	.74	12 (4)	22 (7)	276 (89)

Despite the large numbers of participants who found the orientation helpful, 18% percent (66 of 374) of subjects said that VR navigation (a skill they learn during orientation) was challenging. Furthermore, 26% (98 of 374) believed they needed more time to acclimate to the VR environment before engaging the MCI-VR subway station (Table 2).

### Contribution to learning

While the instructional program designed for use with the MCI-VR system was not specifically intended to be a summative performance assessment, we asked participants about their views regarding the accuracy of the system generated score and whether their encounter performance was a valid assessment of their first responder skills. Both EMS and medical professionals responded similarly to these items, with about three-quarters of participants saying that the score was accurate (77%, 288 of 374) and that it was a valid assessment of their skills (74%, 276 of 374) (Table 3). However, EMS professionals rated the value of feedback and practice with the system significantly lower for making them more effective as a first responder (*P* ≤ 0.001, es = 0.55). The Cohen’s D effect sizes for these differences in mean ratings were considered intermediate in size.

**Table 3:** Contribution to learning. Means (Mn), standard deviations (SD), frequencies and percentages, along with *p*-values for independent t-tests (with Cohen’s d effect sizes (es)), that compared EMS (*N* = 323) to Medical (*N* = 51) professionals on how well the VR system contributed to their learning

Item	Profession	N	Mn	SD	1–2	3	4–5
The MCI-VR generated score accurately reflected of my performance	EMS	323	3.94	.72	11 (3)	57 (18)	255 (79)
	MED	51	3.71	.90	6 (12)	12 (24)	33 (64)
Feedback from MCI-VR will help me improve as a first responder.* (es = .55)	EMS	323	4.31	.59	3 (1)	13 (4)	307 (95)
	MED	51	4.63	.49	0	0	51 (100)
Practicing in the MCI-VR simulator would make me a more effective first responder.* (es = .52)	EMS	323	4.17	.71	6 (2)	41 (13)	276 (85)
	MED	51	4.53	.54	0	1 (2)	50 (98)
My MCI-VR performance was a valid assessment of my current skill as a first responder	EMS	321	3.88	.82	18 (6)	62 (19)	241 (75)
	MED	51	3.78	.83	4 (8)	12 (23)	35 (69)

Notes: Learning was evaluated on a 5-point Likert Response Set: (1 = Strongly Disagree, 2 = Disagree, 3 = Disagree/Agree Equally (D/A =), 4 = Agree, 5 = Strongly Agree). Percentages are based on numbers within professional group (ie. row percentages). Adjusted *P*-values for significance were: k/*P* = .05/5 = .01

^*^ P ≤ .001; es = Cohen’s D effect sizes, which can be interpreted as: .2 to .4 = small, .5 to .7 = intermediate, and ≥ .80 = large [28]

### Future potential of MCI-VR for training first responders

Participant’s general response to the MCI-VR Simulation encounter was positive, with nearly all stating that they would recommend this experience to other first responders (96%, 360 of 374) and assigning it a letter grade of “A” for excellent or “B” for good. (93%, 349 of 374) (Table 4). Despite these positive ratings, only 74% (277 of 374) viewed the MCI-VR training as effective as live training.

**Table 4:** Perceived utility of the VR system for the future. Means (Mn), standard deviations (SD), frequencies and percentages along with independent t-test results (with Cohen’s d effect sizes (es)), that compared EMS (*N* = 323) to Medical (*N* = 51) professionals on the value or utility of the VR system for training in the future

**Item**	**Profession**	**N**	**Mn**	**SD**	**1–2**	**3**	**4–5**
The MCI-VR training was as effective as live training	EMS	322	3.91	.88	27 (8)	51 (16)	244 (76)
	MED	50	3.82	.98	6 (12)	11 (22)	33 (66)
I would recommend this experience to other first responders (or those interested in becoming one).* (es = .45)	EMS	324	4.55	.58	0	14 (4)	310 (96)
	MED	50	4.80	.40	0	0	50 (100)
	**Profession**	**N**	**Mn**	**SD**	**D-F**	**C**	**A-B**
Overall grade I would assign the VR Simulator a grade of:	EMS	319	4.51	.63	2 (1)	17 (5)	300 (94)
	MED	51	4.67	.55	0	2 (4)	49 (96)

Notes: Ratings of effectiveness and recommendations were performed with a 5-point Likert Response Set: (1 = Strongly Disagree, 2 = Disagree, 3 = Disagree/Agree Equally (D/A =), 4 = Agree, and 5 = Strongly Agree). Overall ratings were assigned using letter grades: (F = 1, D = 2, C = 3, B = 4, and A = 5) Percentages are based on numbers within professional group (ie. row percentages). Adjusted *P*-values for significance were: k/*P* = .05/3 = .02

^*^P ≤ .001; es = Cohen’s D effect sizes, which can be interpreted as: .2 to .4 = small, .5 to .7 = intermediate, and ≥ .80 = large [28]

### Summative content analysis

Three-quarters of participants provided comments about strengths of the MCI-VR System (75%, 279 of 347), while fewer than half (49%, 184 of 347) provided suggestions for improving the program.

#### Strengths of the MCI-VR system

Five key themes about strengths of the MCI-VR program were identified by the content analysis: realism, interactivity, and the opportunities to practice triage and treatment skills, decision making, and managing multiple patients (Table 5). The most common comment was about the realism of the simulation, some specifically about the realism of the virtual patients: *“I thought the patient responses/panic even when they were “minimal” was a realistic portrayal of the emotional influence that patients have on providers despite the severity of injuries.”* Several participants recognized the value of the MCI-VR system, including some who appreciated the opportunity to practice without having to risk harm to “real” patients: “*Able to make mistakes without hurting real people.*” A number of participants mentioned the interactivity of the MCI-VR system and the patients within as being key to learning and practicing triage and treatment skills: “*The physical aspect of interacting with patients, treating and assigning them tags was really helpful,” “This was a good refresher and helped remember what equipment is needed.”* Participants also remarked about the importance of experience with triage decision making. Specifically, the simulation pushed participants to *“manage an overwhelming situation and critically think while staying focused.”* Finally, several participants recognized that one key advantage of the VR simulation experience was the opportunity to manage multiple patients collectively instead of one at a time like in tabletop exercises: *“I enjoyed having to manage multiple patients in rapid sequence. This gave me the ability to go from one patient to the next. Other trainings like this present one standardized patient at a time and those patients may not be trained to act or behave appropriately when injured in that manner.”*

**Table 5:** Top themes and illustrative comments derived from the thematic analysis of participant comments about the STRENGTHS of the MCIVR simulation

Theme	Counts	Illustrative Comment(s)
Realistic	*N* = 99	*“Gives you the real-life experience of an MCI without the actual emergency. This also give you real-time decision-making skills.”* *“It's hard to actually recreate a mass casualty. This is a pretty good replacement.”* *“I thought the patient responses/panic even when they were “minimal” was a realistic portrayal of the emotional influence that patients have on providers despite the severity of injuries. I also thought the injuries like the amputation were pretty realistic.”* *“Realistic situation including dim lighting and background noise, patients with a variety of injuries and levels of responsiveness.”* *“It was good as it is. Nice to have something that is close to real life and have to do things and not just verbalize the treatments.”*
Practice with triage and treatment skills	*N* = 32	*“Good practice without needing real people for the scenario.”* *“Being able to use equipment and having patients respond.”* *“Able to make mistakes without hurting real people.”* *“You really get the feel of what it's like to have the pressure of deciding what to do first.”* *“This was a good refresher and helped remember what equipment is needed.”*
Triage Decision Making	*N* = 30	*“Having to assess each patient for myself, having to sort patients and move around to them.”* *“Helpful to go through triage tags and get a feel for the actions needed.”* *“The practice of following the algorithm in real time as a kinestheic learner. Also being able to review after.”* *“Doing the steps of the triage and making me perform critical thinking.”* *“How to manage an overwhelming situation and critically think while staying focused.”*
Interactivity of the Simulator	*N* = 20	*“The physical aspect of interacting with patient, treating and assigning them tags was really helpful.”* *“Speaking to clear the walking wounded out of the way. Made me remember to take a pulse on every victim.”* *“Patients followed commands and all conditions responded to treatment.”* *“Communicating with patients and seeing the wounds.”* *“The patient responsiveness to verbal commands was impressive.”*
Managing Multiple Patients	*N* = 13	*“I enjoyed having to manage multiple patients in rapid sequence. This gave me the ability to go from one patient to the next. Other trainings like this present you with one standardized patient at a time and those patients may not be trained to act or behave appropriately when injured in that manner.”* *“Rapid triaging of multiple patients with varying injuries.”* *“The number of obviously injured patients. It’s good to feel overwhelmed.”* *“Several different patients needing attention necessitating triage.”* *“The number of patients and they were all calling for help.”*

#### Suggestions for improving the MCI-VR system

Most suggestions for improvement had to do with modifying the system by either improving or adding features (Table 6). Many suggestions, particularly those involving patient interactivity, or injury visibility, were used by programmers to make improvements to the system over the period in which these evaluations were conducted. Some features requested by participants were already built into the system but were not yet made available during their one-time encounter. These include the ability to increase the difficulty of the scenario by increasing the levels of chaos: “*Building on this to add more stressors or distractions would be great,*” and “*Make patient status dynamic during the exercise*.” Other suggestions involved possible features for the future, such as adding tools to the medical kit (chest seals, trauma sheers, or a stethoscope), or building virtual sets that resemble environments within the participant’s jurisdiction. Some participants recognized the value in being able to have more than one participant in the MCI-VR environment at the same time: “*Make it multiplayer so crews can work in concert.*” A substantial number of participants experienced system glitches, such as the system freezing or the VR headset losing connectivity to the laptop computer on which the program runs. Glitches occurred regularly at the beginning of Beta testing but subsided over time. Comments about glitches also declined as problems were fixed. Finally, there were a few participants who felt the need for more time in the orientation to become more adept at using the controllers: “*I needed a little more time familiarizing myself with the controls*”.

**Table 6:** Top themes and illustrative comments derived from the thematic analysis of participant SUGGESTIONS FOR IMPROVING the MCI-VR System

**Theme**	**Counts**	**Illustrative Comment(s)**
Modify system by improving features	*N*=52	*“The one thing is it would be great if patients were a bit more interactive in their responses so that trainees could practice asking other questions and getting answers.”* *“Better graphics but these weren't bad by any means.”* *“Need patients to respond quicker when asked questions.”* *“Some patients did not respond well to voice commands until the second or third request.”* *“Make it easier to see chest injury*. *Unclear if patient had burn or crushing chest injury - which influenced management.”* *“The needle decompression didn't work.”*
Modify system by adding features	*N*=49	*“Add more scenarios, like a mass shooting.”* *“Make it multiplayer so crews can work in concert.”* *“Add more responders or the next step in setting up transport from the collection point.”* *“Add more tools to the med kit like trauma sheers, chest seals, and stethoscope.”* *“Give us option to remove clothing on patients to reveal injuries.”* *“Dropping in actual buildings in our jurisdiction.”* *“Add chest seal for sucking chest wounds. Combat gauze is not a good intervention for packing chest and abdominal wounds. It is more well suited for inguinal injuries (which were not represented in this blast scenario.”* *“Have a visible time clock counting down during the encounter.”* *“Make patient status change during the exercise.”*
Improve system reliability	*N*=33	*“There were some glitches and lags with the system, like how the kit was often outside of my field of vision, so it was tough to grab it when needed.”* *“Just work out some of the bugs and limitations. For example, treating a patient with needle decompression that did not get recognized.”* *“Remedy the glitches, like disappearing medical kit.”* *“The lag between the device and the computer running it was distracting one or two times.”* *“There were some glitches and lags with the system, like how the kit was often outside of my field of vision, so it was tough to grab it when needed.”*
Make scenario more chaotic/challenging	*N*=23	*“Background noise and radio traffic would make it more challenging.”* *“Building on this to add more stressors or distractions would be great.”* *“More chaos-distractions, noise, screaming.”*
Improve orientation/preparation for VR	*N*=22	*“I needed a little more practice opening med kit and moving. But was not an issue for very long.”* *“A little more time familiarizing with the controls”* *“More acclimation before event”* *“More time to prepare for those have never used it.”* *“I did not realize the gray-expectant tag was in the kit until the feedback at the end.”*
Make system more available	*N*=13	*“Do it again in 6 months or a year to see if or how we've improved.”* *“Make it easy for use individually outside of work.”* *“More opportunities to try it.”*

## Discussion

Participants of the SALT Training with the MCI-VR System would recommend this experience to others and graded the experience as an “A” for excellent or “B” for good. The system was equally received as an effective training experience by both prehospital and hospital care providers, regardless of prior experience with triage or disaster training. Both groups credited the feedback from the activity for improving their first responder skills and believed that continued practice with the system would make them more effective first responders. While some participants commented on the need for more time to acclimate to the VR environment, we found no statistical differences in ratings of the system between those who owned VR systems and those who did not. Participants rated the system’s realism and interactivity highly, and reinforced these ratings with similar comments about how these were strengths of the system.

Three-quarters of our participants considered the MCI-VR training to be as effective as live, large-scale exercises, which may be more realistic, but are expensive, resource intense, requiring substantial planning and amounts of space. This finding suggests that VR for disaster response training is a viable option and could provide EMS agencies more flexibility in offering their first responders opportunities to learn and practice triage and treatment skills in realistic recreations of mass casualty incidents.

Virtual reality technology has gradually improved over the past ten years to become more immersive and realistic. Earlier versions of VR technology for training disaster response skills, such as Second Life, resembled computer versions of tabletop drills by presenting content on 2D computer screens. Participants interacted with these versions by controlling avatars through keyboards and mouse controls [29, 30]. VR became more immersive with the use of computer automated virtual environments (CAVEs), which used wall projection and liquid crystal glasses to create simulated disaster environments [31, 32]. CAVEs immersed subjects in the simulation rather than watching an avatar on a computer screen. The major drawback to these CAVE systems was that they employed expensive technology that limited their use to institutions with simulation centers and large simulation budgets [33].

Recent advances in VR technology now allow for the creation of realistic, highly immersive experiences at a much lower cost than these earlier systems. Our simulation used a MetaQuest 2 VR head-mounted display with controllers that allowed for individual tracking, tactile pulse checks, and highly immersive simulations. As such, VR simulations that were previously restricted to very expensive systems in large simulation centers can now be created at significantly less cost and be made accessible to a wider variety of organizations from university or medical school training programs to local fire, rescue, and EMS agencies. Moreover, assessments can be automated and built into the simulation, further increasing the utility of newer VR approaches.

Our VR simulation illustrates the practicality of this technology for disaster and mass casualty training. The MCI-VR system was well received by a variety of learners at different levels of training and did not rely on prior computer gaming experience, nor prior experience with disaster training or drills. Literature on optimal ways to prepare learners for VR environments was limited, so we designed our own brief orientation to help learners transition. The responses of our learners made clear that for most, a brief (approximately 10 min) guided tutorial and orientation was an effective way to prepare them for the actual simulation. This tutorial allowed the most novice VR learners to focus on the triage and treatment of patients without having to worry about the “knobology” of controlling the VR system.

One aspect of the training praised by learners was the value of the expert debriefing. Although the system generated a score report that could be used by the learner, we chose to provide formative feedback by reviewing these reports with participants in person. Our customizable system provided a structure that could leverage simulation-based mastery learning to gradually improve a learner’s mastery of triage and treatment skills over time [34]. Furthermore, this system provides a living laboratory for studying and evaluating triage protocol efficacy and the performance of learners from various backgrounds.

### Limitations

This project examined participants’ evaluation of SALT triage training using the MCI-VR system, which is analogous to their satisfaction with the experience. Subsequently, we are not able to conclude that the training improved participant triage and treatment performance. Nor are we able to conclude that patient outcomes would improve because of improved first responder skills. Future research will be needed to attain these higher-level objectives. Although we screened for motion sickness prior to entry into the MCI-VR system, we did not ask participants about the impact of the system on them, either physiologically or psychologically, after the encounter [35]. While we assume from their positive evaluations that participants responded to the simulation similarly to a real-life incident, we were not able to verify this assumption.

### Future research

Evaluation feedback has been used to improve the MCI-VR simulator including the expansion of the tools available in the medical kit and responsiveness of the virtual patients. Respondents have encouraged further development of the system, suggesting the inclusion of other disaster scenarios, and increasing the capacity of participants in the system for developing teamwork and communication. We are currently analyzing system generated data to investigate participant’s performance: such as time to hemorrhage control, and accuracy and efficiency in triaging and treating the scene. Further, we are planning subgroup analyses to compare the performances of first responders from varied training backgrounds (medical students, residents, paramedics and EMTs). Finally, we plan to leverage the VR system for studying human factors such as stress and their relationship to first responder performance.

## Conclusions

Our VR simulator is an automated, customizable, fully immersive virtual reality system for training and assessing personnel in the proper response to a mass casualty incident. Participants perceived the encounter as realistic and effective for training, regardless of their prior level of training or their experience using virtual reality. The MCI-VR simulator was demonstrated to be a viable training alternative to current methods of training disaster response.

## Supplementary Information

Supplementary Material 1.

## Data Availability

The datasets used and/or analysed during the current study are available from the corresponding author upon reasonable request.

## Abbreviations

VR: Virtual Reality
SALT: Sort, Assess, Lifesaving interventions, and Treatment and/or Transport
EMS: Emergency Medical Service
U.S.: United States
MCI: Mass Casualty Incident
CDC: Center for Disease Control
EM: Emergency Medicine
HMD: Head mounted display
LSI: Life saving intervention
MCI-VR: Mass casualty incident virtual reality
P: Probability under the null hypothesis
K: Number of pair-wise comparisons
Es: Effect size
EMT: Emergency Medical Technician
N: Number of subjects
Mn: Mean
SD: Standard Deviation
D/A: Disagree/Agree Equally
AI: Artificial Intelligence
