# Supplementary material for: Learner evaluation of an immersive virtual reality mass casualty incident simulator for triage training
Source: BMC Digit Health. 2024 Sep 16;2(1):56. doi: 10.1186/s44247-024-00117-5 (PMC11402856; doi:10.1186/s44247-024-00117-5)
Supplement: Supplementary file 1 — Supplementary Material 1. [file 44247_2024_117_MOESM1_ESM.docx]

**Appendix A. Post-MCI-VR Encounter Questionnaire**

The purpose of this questionnaire is three-fold. First, we are requesting formative feedback about your experience with the mass-casualty incident virtual reality (MCIVR) simulator. We will use this feedback for improving the system. Second, we are interested in your assessment of the simulator for its potential to train first responders. Finally, we would like to know a little about you and your experience with virtual reality and disaster preparedness training.

**Feedback about Your MCIVR Experience**

Please select the response that best matches your opinion. Use the following key:

**SD=Strongly Disagree; D=Disagree; NDA=Neither Disagree nor Agree; A=Agree; SA=Strongly Agree**

1. The virtual reality simulation exercise was realistic.

2. I was adequately prepared to enter the MCI-VR subway station.

3. I needed more time to acclimate to the VR before entering the MCI-VR subway station.

4. The orientation helped me to master navigation through the MCI-VR subway station.

5. The virtual patients were realistic.

6. The virtual patients responded to my commands.

7. Navigation throughout the MCI-VR subway station was challenging.

8. The medical kit contained everything I needed.

9. I found it easy to use the instruments from the medical kit.

10. I would recommend this experience to other first responders (or those interested in becoming one).

11. Please name 1-2 attributes of this VR experience that were particularly effective at preparing you to become an effective first responder.

12. Please name 1-2 things that we might do to improve this virtual reality experience.

**Training Potential of the MCIVR Simulator**

Please select the response that best matches your opinion. Use the following key:

**SD=Strongly Disagree; D=Disagree; NDA=Neither Disagree nor Agree; A=Agree; SA=Strongly Agree**

1. The MCI-VR generated score accurately reflected my performance as a first responder.

2. Feedback I received from the MCI-VR experience will help me to improve my performance as a first responder.

3. Practicing in the MCI-VR simulator would make me a more effective first responder.

4. The MCI-VR training was as effective as live training.

5. My MCI-VR performance was a valid assessment of my skill as a first responder.

Please select the response that best matches your opinion. Use the following key:

**F=Poor; D=Fair; C=Satisfactory; B=Good; A=Excellent**

1. Overall, the grade I would assign the virtual reality simulator is:

**Prior Experience**

Please select the response that best matches your opinion. Use the following key:

**SD=Strongly Disagree; D=Disagree; NDA=Neither Disagree nor Agree; A=Agree; SA=Strongly Agree**

1. I am a computer gaming enthusiast.

2. I own a virtual reality (VR) system.

3. I consider myself a seasoned first responder.

4. I have completed the SALT Triage Certificate Training Course (Sponsored by a national or statewide agency).

5. I have completed triage training other than SALT Triage Training before.

6. I have completed disaster response training such as those offered by the American Red Cross, FEMA, or the Community Emergency Response Team (CERT).

How many disaster drills (or simulated mass casualty events with live actors) have you participated in before today. (Please enter a whole number).

What is your current role (choose all that apply):

| M-3 medical student | Emergency medical technician |
| --- | --- |
| M-4 medical student | Intermediate EMT |
| EM resident | Advanced EMT |
| EM/IM resident | Paramedic |
| Other resident | Military medic |
| EM fellow | Tactical EMS instructor |
| EM faculty | Tactical paramedic |
| PEM faculty | Other: Please specify |
